# Jugular arginine supplementation increases lactation performance and nitrogen utilization efficiency in lactating dairy cows

**DOI:** 10.1186/s40104-018-0311-8

**Published:** 2019-01-21

**Authors:** Luoyang Ding, Yizhao Shen, Yifan Wang, Gang Zhou, Xin Zhang, Mengzhi Wang, Juan J. Loor, Lianmin Chen, Jun Zhang

**Affiliations:** 1 College of Animal Science and Technology, Yangzhou University, Yangzhou, 225009 Jiangsu People’s Republic of China; 2 School of Agriculture and Environment, The University of Western Australia, Perth, WA 6009 Australia; 3 Clinical Medical School, Southeast University, Nanjing, 210009 Jiangsu People’s Republic of China; 4 Department of Animal Sciences and Division of Nutritional Sciences, University of Illinois, Urbana, IL 61801 USA

**Keywords:** Amino acid transporters, Arginine, Lactation, Milk protein

## Abstract

**Background:**

Enhancing the post-ruminal supply of arginine (Arg), a semi-essential amino acid (AA), elicits positive effects on milk production. Our objective was to determine the effects of Arg infusion on milk production parameters and aspects of nitrogen (N) absorption and utilization in lactating dairy cows. Six lactating Chinese Holstein cows of similar body weight (508 ± 14 kg), body condition score (3.0 ± 0), parity (4.0 ± 0), milk yield (30.6 ± 1.8 kg) and days in milk (20 ± 2 d) were randomly assigned to 3 treatments in a replicated 3 × 3 Latin square design with 21 d for each period (1 week for infusion and 2 weeks for washout). Treatments were 1) Control: saline; 2) Arg group: saline + 9.42 g/L *L*-Arg; 3) Alanine (Ala) group: saline + 19.31 g/L *L*-Ala (iso-nitrogenous to the Arg group). Milk production and composition, dry matter intake, apparent absorption of N, profiles of amino acids (AA) in blood, urea N in urine, milk, and blood, and gene expression of AA transporters were determined.

**Results:**

Compared with the Control or Ala group, the infusion of Arg led to greater expression of AA transporters (*SLC7A2* and *SLC7A8*) and apparent uptake of free AA in the mammary gland, and was accompanied by greater milk yield, milk protein yield and milk efficiency (calculated by dividing milk yield over feed intake), together with lower concentration of urea N [regarded as an indicator of N utilization efficiency (NUE)] in blood and milk. Furthermore, in the cows infused with Arg, the NUE was higher and the concentration of urea N in urine was lower than those in the Ala group, although no differences were detected in NUE and urea N in urine between the Control and Arg group. The infusion of Ala had no effect on those indices compared with the Control.

**Conclusions:**

Overall, enhancing the post-ruminal supply of Arg via the jugular vein had a positive effect on the synthesis of milk protein at least in part by increasing gene expression of some AA transporters and uptake of free AA by mammary gland.

## Background

Current intensive livestock management systems encourage the inclusion of a large amount of crude protein in diets to support higher rates of production, a practice that often decreases nitrogen utilization efficiency (NUE) and increases production costs [1]. Regardless of stage of lactation and level of production, it is estimated that almost 72% of dietary N is excreted in feces and urine [2]. Excess N excretion creates environmental problems including the release of ammonia to the air and nitrate contamination of soil and groundwater [3, 4]. Therefore, the search for approaches to decrease output of N in manure and into the atmosphere continues [5].

The most direct and effective way to decrease manure N is to reduce dietary N concentration. As reported previously [3, 6], fecal and urinary N in dairy cows decreases significantly with the reduction of dietary crude protein (CP) content. Despite these benefits, a decrease in dietary CP could have negative effects on production efficiency. For example, a decrease of CP in diets from 18% to 14% decreased milk yield from 26.0 to 22.5 kg/d and milk protein yield from 800 to 710 g [7]. Clearly, one way to alleviate these discrepancies is the improvement of NUE, for instance, by increasing post ruminal digestibility or providing a pattern of absorbed amino acids (AA) that closely matches the AA requirements for milk synthesis [8].

Some recent studies demonstrated that the addition of limiting AA (methionine, lysine, histidine) for milk protein synthesis in diets increased milk volume and milk protein production in dairy cows [9–11]. The supplementation of Arg (a conditionally essential AA) in diets was also reported to enhance litter weight gain of primiparous sows and milk production of nursing sows [12]. From a mechanistic standpoint, Arg supply enhances casein synthesis in bovine mammary epithelial cells (BMEC) and protein synthesis in muscle of neonatal pigs through its effect on intracellular signalling pathways [13, 14]. In the present study, we sought to test the general hypothesis that enhancing the supply of Arg to lactating cows would increase milk yield and enhance the synthesis of milk protein at least in part by increasing NUE. Specific objectives were to assess the regulatory roles of greater Arg supply through jugular infusions on milk production parameters, aspects of N absorption and utilization, and mammary gene expression of amino acid transporters.

## Methods

### Experimental animals and management

Six lactating Chinese Holstein cows of similar body weight (508 ± 14 kg), body condition score measured with a 5-point scale (3.0 ± 0) [15], parity (4.0 ± 0), milk yield (30.6 ± 1.8 kg) and days in milk (20 ± 2 d) were selected for the present study. Prior to the infusion period, indwelling catheters (L13712, Jiangxi Huali Medical Instrument Company, Ganzhou, China) were placed in the jugular vein and flushed with heparin and physiological saline (150 IU/mL) twice daily during the whole experimental period. Cows were fed the same basal diet formulated according to NRC [16] at the Experimental Farm of Yangzhou University (Yangzhou, Jiangsu, China). The composition and nutrient level of the basal diets are described in Table 1. Cows were fed twice daily (06:00 and 20:00 h), and milked thrice daily (07:00, 15:00 and 23:00 h) with a portable milker (HL-JN02, Chuangpu Machinery Limited Company, Lianyungang, China), and housed individually in a free-stall barn. They had ad libitum access to the TMR and fresh water.

**Table 1:** Ingredient and composition (DM basis) of the basal diets used in this infusion study

Ingredients	%	Nutrients^b^	
Alfalfa	20.90	NE_L_, Mcal/kg	1.59
Chinese wildrye	3.80	CP, %	15.83
Corn silage	24.60	NFC^c^, %	33.11
Corn	27.70	NDF, %	43.63
Cottonseed meal	3.70	ADF, %	25.93
Soybean meal	13.50	EE, %	4.03
DDGS	3.80	Ca, %	0.96
CaHPO_4_	0.30	Total P, %	0.48
NaCl	0.50		
Premix^a^	1.20		
Total	100.00		

^a^The premix provided following per kilogram of diet: CuSO_4_ 25 mg, FeSO_4_·H_2_O 75 mg, ZnSO_4_·H_2_O 105 mg, Co 0.0024 mg, Na_2_SeO_3_ 0.016 mg, vitamin A 12,000 IU, vitamin D_3_ 10,000 IU, vitamin E 25 mg, nicotinic acid 36 mg, choline 1,000 mg

^b^NE_L_ in diet was calculated according to the NE_L_ of ingredients and their percentages; concentrations of the other nutrients were measured values

^c^NFC = 100 – (NDF% + CP% + EE% + Ash%)

### Experiment design

Cows were randomly divided into 3 groups with 2 cows in each group in a replicated 3 × 3 Latin square design with 21 d for each period (1 week for infusion and 2 weeks for washout). Treatments were as follows: 1) Control: saline; 2) Arg group: saline + 9.42 g/L *L*-Arg; 3) Ala group: saline + 19.31 g/L *L*-Ala (iso-nitrogenous to the Arg group). Perfusates were prepared by Cambridge Biological Company (Nanjing, China) in sterile conditions 2 d before the infusion period and stored at 4 °C. The solutions were infused continuously through a peristaltic pump (Longer, Hebei, China) with a speed of 0.5 L/h for 8 h/d (from 06:00 to 14:00 h).

### Sampling and chemical analysis of feed and feces

Within each period during the infusions, the daily amount of diet offered and residual feed for each cow were recorded for calculation of feed intake. Samples of fresh TMR and refusals from each cow were collected daily during the last 3 d of each infusion period. Fecal grab samples were collected 3 times daily (06:00, 13:00 and 20:00 h) and a pool of 100 g feces was made from the combination of each time point. Samples were stored at − 20 °C. Diet ingredients and feces were dried in a forced-air oven (DHG9626A, Jinhong Co., Shanghai, China) at 60 °C and ground to 1 mm particle size for analyses. Dry matter and CP were analyzed by using methods described by Kopelove et al. [17]. Dietary and fecal N content was determined and CP was calculated using the 6.25 factor, in which CP (g/kg) = N (g/kg) ÷ 6.25. Apparent absorption of nitrogen was calculated using the formula as follows:

$$ \mathrm{Apparent}\kern0.17em \mathrm{absorption}\;\left(\%\right)=\left(1\hbox{-} \frac{\mathrm{marker}\ \mathrm{in}\ \mathrm{diet}\%}{\mathrm{marker}\ \mathrm{in}\ \mathrm{faeces}\%}\times \frac{\mathrm{nutrient}\ \mathrm{in}\ \mathrm{faeces}\%}{\mathrm{nutrient}\mathrm{s}\ \mathrm{in}\ \mathrm{diet}\%}\right)\times 100 $$

according to the method of 4 mol/L HCl acid-insoluble ash [18].

### Sampling and analysis of blood

In the last day of the infusion period, samples of blood from the mammary vein and coccygeal artery [19] were collected into 10 mL heparinized vacuum tubes (Becton Dickinson and company, Franklin Lakes, NJ, USA) every 3 h from 06:00 to 15:00 h. Samples were then centrifuged (10 min, 1800×*g*) immediately, and the plasma was aliquoted. An extra blood sample from the coccygeal artery was collected into 10 mL serum tubes (Becton Dickinson Vacutainer System), and centrifuged (10 min, 2810×*g*) 2–3 h after collection to isolate the serum. In order to more precisely sample the blood from coccygeal artery, we first placed the needle into the blood vessel and determined visually if the needle puncture the artery (bright red blood) or vein (dark red blood). Once it was verified that the needle had punctured the artery, we proceeded with sampling. The serum and plasma samples were stored at − 20 °C prior to analysis of biochemical indices. Equal amounts of plasma collected at 06:00, 09:00, 12:00 and 15:00 h from each cow were mixed and deproteinized prior to analysis of AA concentrations with an L-8900 Amino Acid Analyzer (Hitachi High-Technologies, Dallas, TX, USA). The standard AA mixture solution was purchased from Wako Pure Chemical Industries (Amino Acids Mixture Type H, Osaka, Japan). In addition, serum samples from different time points were also mixed for the analysis of blood urea N with an Urea Assay Kit (C013–2, Jiancheng Biogineering Institute, Nanjing, China).

### Production data collection and milk sample analysis

Milk production was recorded at each milking and sampled on the last 2 d of each infusion period (the 6^th^ and 7^th^ day). Milk samples were collected 3 times a day and a pool made from each sample in proportion to milk yield at each milking. A subsample of milk was used for analysis (Bentley FTS/FCM 400 Combi; Bentley Instrument, Chaska, USA). Another subsample was centrifuged at 2810×*g* at 4 °C for 10 min to remove fat before measuring urea N with a Urea Assay Kit (C013–2, Jiancheng Biogineering Institute, Nanjing, China). Milk protein N content was calculated after determination of protein using a 6.38 factor, in which N (g/kg) = protein (g/kg) × 6.38. The NUE was calculated by dividing milk protein N by dietary N intake.

### Urine sampling and detection of urea nitrogen

Total urine was collected using a simple urine cup method [20], weighed, and 5% of total volume sampled on the last 2 d of each infusion period. 50% H_2_SO_4_ was added to the collection bucket before sampling urine to minimize volatilization. After the collection of urine, the pH of urine samples was adjusted to 2 and 4 prior to storage at 4 °C [21]. The concentration of urea N was measured with a Urea Assay Kit (C013–2, Jiancheng Biogineering Institute, Nanjing, China).

### Mammary gland biopsy and PCR for gene expression

*Mammary gland biopsy*. Mammary gland tissue was sampled at the end of each infusion period by using a published biopsy method [22]. The cows were first given a small dose of general anesthetic (0.025 mg/kg BW Xylazine, 20 mg/mL) prior to biopsy. Tissue was then isolated and placed into 1.5 mL cryovials and frozen immediately in liquid N prior to storage at − 80 °C until RNA extraction.

*RNA extraction and reverse transcription*. Frozen mammary tissue was quickly minced, weighed (0.5–1.0 g), and placed in ice-cold TRIzol (15596018, ThermoFisher, Carlsbad, USA) as described previously [23]. Total RNA was precipitated with isopropanol, washed with 70% ethanol, and resuspended in UltraPure^TM^ DNase/RNase-Free Distilled Water (10977015, ThermoFisher, Carlsbad, USA). The integrity of RNA was assessed by agarose gel electrophoresis by analyzing 28S and 18S rRNA subunits. The concentration of RNA was measured with a Nanodrop spectrophometer (ThermoFisher). A portion of the RNA was then diluted to 100 ng/μL before reverse-transcription with the High-capacity cDNA Reverse Transcription Kit (4368813, Applied Biosystems, Carlsbad, USA). Each cDNA was synthesized in a 20-μL reaction (including 2 μL 10× RT buffer, 0.8 μL 100 mmol/L dNTP Mix, 2.0 μL 10× RT Random Primers, 1.0 μL MultiScribe^TM^ Reverse Transcriptase, 1 μL Rnase inhibitor, 3.2 μL Nuclease-free H_2_O and 10 μL RNA sample). The mixture was incubated at 25 °C for 10 min, 37 °C for 120 min, 85 °C for 5 min and kept at 4 °C for RT-PCR analysis.

*Primer sequence of selected genes*. Glyceraldehyde-3-phosphate dehydrogenase (*GAPDH*) and ß-actin were selected as internal control genes. Furthermore, 6 genes associated with amino acid transport were selected: solute carrier family 7 member 1 (*SLC7A1*), solute carrier family 7 member 2 (*SLC7A2*), solute carrier family 7 member 5 (*SLC7A5*), solute carrier family 7 member 6 (*SLC7A6*), solute carrier family 7 member 7 (*SLC7A7*) and solute carrier family 7 member 8 (*SLC7A8*). Primer sequences of genes were the same as that in the previous study [24] and are listed in Table 2.

**Table 2:** Primers of genes for real-time PCR analysis

Gene name	ID number	Sequence (5’to3’)	Product size, bp ^a^	Annealing temperature, °C
*SLC7A1*	NM_001135792	F: TCAACCAGCCTCCTAGCACT	147	60
		R: GGCACCTTGAATGAGAGCTT		
*SLC7A2*	XM_010820288	F: CTCGCTCCTCCGTGATAAAT	168	63
		R: ATTCAGCGATCACCTCCATT		
*SLC7A6*	NM_001075937	F: GCCTTTGGTTCAGGGTATGC	128	60
		R: CTGGATAGGTGCCAGCTT		
*SLC7A7*	NM_001075151	F: GGAGCCTCGACTCACTTTGA	133	60
		R: GTCCCTCATGTTGAGCACAG		
*SLC7A5*	NM_173892	F: CTGATGGAGTATGCGAAGCA	164	55
		R: GGGTCCTGGGTCTTTGTGTA		
*SLC7A8*	NM_001034428	F: CTGCGAACTCATCAGAACCA	134	63
		R: CTCCAGCAGCCTTTCAGAGT		
*ß-actin*	NM_173979.3	F:ACTGTTAGCTGCGTTACACCCTT	190	62
		R: TGCTGTCACCTTCACCGTTCC		
*GAPDH*	NM_001034034.1	F: GGTCACCAGGGCTGCTTT	176	59
		R: CTGTGCCGTTGAACTTGC		

Note: All the primers used in this experiment were synthesized in Invitrogen (Nanjing, China)

^a^bp = bases of pairs

Genes: GAPDH = glyceraldehyde-3-phosphate dehydrogenase; SLC7A1 = solute carrier family 7 member 1; SLC7A2 = solute carrier family 7 member 2; SLC7A5 = solute carrier family 7 member 5; SLC7A6 = solute carrier family 7 member 6; SLC7A7 = solute carrier family 7 member 7; SLC7A8 = solute carrier family 7 member 8

*RT-PCR analysis.* RT-PCR analysis was performed with the Power SYBR® Green PCR Master Mix (4367659, Applied Biosystems, Carlsbad, America) in a 20-μL reaction mix (10 μL 2× Fast SYBR® Green Master Mix, 0.8 μL of 10 μmol/L forward and reverse primers, 1 μL cDNA template and 7.4 μL RNase-free water). Each sample was run in triplicate in the ABI Prism 7500 Detection Instrument (Applied Biosystems) using the followed protocol: 30 s at 95 °C, 10 s at 95 °C, 20 s annealing temperature, and 30 s at 72 °C for 40 cycles. The same conditions were performed on an equal amount of RNase-free water as a negative control. Gene expression was calculated using the 2^-ΔΔCt^ method [25].

### Statistical analysis

Data were analyzed using the general linear model procedures of SPSS 16.0:

$$ {\mathrm{Y}}_{\mathrm{i}\mathrm{jk}}=\upmu +{\mathrm{T}}_{\mathrm{i}}+{\mathrm{P}}_{\mathrm{j}}+{\mathrm{C}}_{\mathrm{k}}+{\mathrm{e}}_{\mathrm{i}\mathrm{jk}}, $$

where Y_ijk_ = response variable value of the kth cow subjected to the i^th^ treatment in the j^th^ period, μ is the grand mean, e_ijk_ = the random error, T_i_ = fixed effect of the i^th^ treatment (i = Control, Arg group and Ala group), P_j_ = random effect of the j^th^ period (j = 1, 2 and 3) and C_k_ = random effect of the k^th^ cow. Treatment differences were determined by the Tukey multiple comparison test and were considered significant if *P* < 0.05.

## Results

### Effects of arginine infusion on feed intake and production parameters

Table 3 reports the effects of different treatments on feed intake and production parameters. The milk yield and milk efficiency in the Arg group were higher than that in Control (*P* = 0.03 and *P* = 0.03 respectively) or Ala group (*P* = 0.03 and *P* = 0.048 respectively). However, no differences in milk yield (*P* = 0.60) and milk efficiency (*P* = 0.43) were detected between Control and Ala group. Furthermore, the infusion of Arg increased the content and yield of protein in milk compared with Control (*P* = 0.001 and *P* = 0.01 respectively) or Ala group (*P* = 0.049 and *P* = 0.04 respectively), while there were no differences in milk protein content (*P* = 0.60) and yield (*P* = 0.08) between Control and Ala group. Furthermore, the daily feed intake, the concentrations and yields of milk fat or solids nonfat were also not affected by treatments.

**Table 3:** Effect of arginine infusion on the feed intake and production parameters of lactating dairy cows

Items	Treatments^c^			SEM	*P*-value
	Control	Arg group	Ala group		
Feed intake, kg DM/d	21.69	22.33	22.82	0.90	0.48
Milk yield, kg/d	25.45^b^	28.16^a^	25.95^b^	0.92	0.03
Milk fat, %	4.42	4.19	4.09	0.14	0.09
Milk protein, %	3.04^b^	3.17^a^	3.11^b^	0.03	< 0.01
Solids nonfat, %	8.81	8.73	8.75	0.21	0.93
Milk fat yield, g/d	1125.88	1179.80	1064.26	61.92	0.22
Milk protein yield, g/d	774.97^b^	893.95^a^	805.80^b^	31.38	0.01
Solids nonfat yield, g/d	2241.28	2458.33	2271.29	101.66	0.11
Milk efficiency^d^	1.12^b^	1.30^a^	1.17^b^	0.06	0.03

^a,b^Different superscripts within a column represent significant differences (*P* < 0.05)

^c^Control, saline; Arg group, saline + 9.42 g/L *L*-Arg; Ala group: saline + 19.31 g/L *L*-Ala

^d^Milk efficiency: Milk yield/Feed intake

### Effects of arginine infusion on absorption and utilization of nitrogen

The effects of different treatments on absorption and utilization of nitrogen are described in Table 4. Compared with Control (*P* = 0.01) or Ala group (*P* = 0.04), the production of protein N in milk in cows infused with Arg was greater, but no difference in the yield of milk protein N was found between Control and Ala group (*P* = 0.60). Furthermore, the infusion of Arg increased the NUE compared with those infused with alanine (*P* = 0.04). No difference in the NUE was detected between Control and Arg group (*P* = 0.06) or Ala group (*P* = 0.97). In addition, there were no differences in the dietary N intake, fecal N, absorption of dietary N intake and total N intake across treatments.

**Table 4:** Effects of arginine infusion on the absorption and utilization of nitrogen in lactating dairy cows

Items	Treatments^c^			SEM	*P*-value
	Control	Arg group	Ala group		
Dietary N intake, g/d	485.74	500.21	511.10	20.25	0.48
Fecal N, g/d	152.36	137.68	138.53	11.6	0.39
Absorption of dietary N, %	68.53	72.38	72.91	2.36	0.17
Milk protein N^d^, g/d	121.47^b^	140.12^a^	126.30^b^	4.92	0.01
NUE^e^, %	25.01^ab^	28.20^a^	24.69^b^	1.25	0.03

^a,b^Different superscripts within a column represent significant differences (*P* < 0.05)

^c^Control, saline; Arg group, saline + 9.42 g/L *L*-Arg; Ala group: saline + 19.31 g/L *L*-Ala

^d^The daily production of milk protein N was calculated after determination of milk protein using a 6.38 factor, in which N (g/d) = protein (g/d) × 6.38

^e^NUE (nitrogen utilization efficiency) = milk protein N/dietary N intake

The effects of treatments on urea N in serum, urine and milk of cows are reported in Table 5. The concentration of urea N in serum and daily quantity of milk urea N in cows infused with Arg were lower compared with Control (*P* = 0.01, *P* = 0.004 respectively) or Ala group (*P* = 0.03, *P* = 0.04 respectively). Furthermore, the quantity of urea N in daily urine in cows infused with Arg was also lower than that in the Ala group (*P* = 0.04). No difference was found in this index between Control and the Arg (*P* = 0.13) or Ala group (*P* = 0.78).

**Table 5:** Effects of arginine infusion on the urea N in serum, urine and milk of dairy cows

Items	Treatments^c^			SEM	*P*-value
	Control	Arg group	Ala group		
Urea N in serum, mmol/L	1.68^a^	1.36^b^	1.63^a^	0.09	0.01
Urea N in urine, g/d	155.85^ab^	131.05^b^	164.08^a^	11.94	0.04
Urea N in milk, g/d	9.42^a^	7.80^b^	8.95^a^	0.42	0.01

^a,b^Different superscripts within a column represent significant differences (*P* < 0.05)

^c^Control, saline; Arg group, saline + 9.42 g/L *L*-Arg; Ala group: saline + 19.31 g/L *L*-Ala

### Effects of arginine infusion on plasma profiles and arteriovenous differences of free amino acids

As shown in Table 6, compared with Control (*P* = 0.03) or Ala group (*P* = 0.02), the infusion of Arg significantly increased Arg concentration in coccygeal plasma. A similar result was also found for the concentration of alanine in arterial plasma which was greater in the Ala group compared with the Control (*P* = 0.01) or Arg group (*P* = 0.049). There were no significant differences in the concentrations of other free AA, total essential AA (TEAA), total non-essential AA (TNAA), and total free AA (TFAA) among different treatments.

**Table 6:** Effects of arginine infusion on free AA concentrations (μmol/L) in plasma of coccygeal artery in dairy cows

Items	Treatments^c^			SEM	*P*-value
	Control	Arg group	Ala group		
Lys	182.23	183.48	176.69	18.15	0.92
Met	65.88	63.30	63.06	11.05	0.96
Leu	322.49	305.12	371.40	43.96	0.32
Ile	187.06	176.20	211.19	23.34	0.34
Arg	195.36^b^	254.45^a^	191.55^b^	20.75	0.02
His	133.21	165.01	126.13	17.40	0.09
Phe	127.03	119.41	150.87	16.24	0.16
Thr	181.08	199.64	152.40	28.53	0.28
Val	285.17	273.02	315.51	27.63	0.31
TEAA^d^	1679.52	1739.62	1758.82	140.56	0.84
Ala	187.04^b^	207.12^b^	253.09^a^	17.65	0.01
Gly	351.45	382.51	333.45	39.15	0.47
Glu	125.56	126.81	123.13	23.50	0.99
Ser	95.58	95.35	80.27	7.35	0.09
Pro	118.20	129.34	139.41	14.00	0.34
Tyr	76.21	94.93	68.44	11.38	0.09
Cys	41.75	52.64	47.25	5.45	0.17
Asp	1.15	1.37	1.04	0.35	0.65
TNAA^d^	996.93	1090.09	1046.09	72.94	0.46
TFAA^d^	2676.45	2829.71	2804.91	160.65	0.60

^a,b^Different superscripts within a column represent significant differences (*P* < 0.05)

^c^Control, saline; Arg group, saline + 9.42 g/L *L*-Arg; Ala group: saline + 19.31 g/L *L*-Ala

^d^TEAA: Total essential amino acids; TNAA: Total non-essential amino acids; TFAA: total free amino acids

No effects on the arteriovenous differences of Lys, Leu, Ile, Phe, Val, Ala, Gly, Ser, Pro, Tyr, Cys, Asp and TNAA were detected among different treatments (Table 7). However, compared with the Control, the infusion of Arg increased the arteriovenous differences of Met (*P* = 0.001), Arg (*P* = 0.01), His (*P* = 0.01), Thr (*P* = 0.01), Glu (*P* = 0.03), TEAA (*P* = 0.001) and TFAA (*P* = 0.02). Furthermore, the arteriovenous differences of Met (*P* = 0.003), Arg (*P* = 0.02), His (*P* = 0.04), Thr (*P* = 0.01), Glu (*P* = 0.002), and TEAA (*P* = 0.003) in the Arg group were greater compared with the Ala group. While the arteriovenous differences of TFAA in Arg group was similar to that in Ala group (*P* = 0.06).

**Table 7:** Effects of arginine infusion on arteriovenous difference in free AA concentrations (μmol/L) of dairy cows

Items	Treatments^c^			SEM	*P*-value
	Control	Arg group	Ala group		
Lys	91.30	100.09	90.13	5.08	0.14
Met	12.99^b^	19.12^a^	14.13^b^	1.26	< 0.01
Leu	84.29	85.27	84.58	4.54	0.98
Ile	43.83	41.33	44.57	2.66	0.46
Arg	61.62^b^	76.51^a^	63.70^b^	4.29	0.01
His	95.68^b^	122.67^a^	103.33^b^	7.08	0.01
Phe	28.58	27.71	24.97	3.74	0.61
Thr	79.82^b^	90.86^a^	79.58^b^	3.27	0.01
Val	71.67	69.68	71.58	4.16	0.87
TEAA^d^	569.79^b^	633.25^a^	576.57^b^	14.36	< 0.01
Ala	136.98	145.09	145.77	10.37	0.65
Gly	229.23	238.24	237.59	19.39	0.88
Glu	60.64^b^	72.16^a^	54.95^b^	4.03	< 0.01
Ser	62.78	66.73	66.51	2.21	0.17
Pro	66.76	64.08	64.58	5.16	0.86
Tyr	26.54	23.79	23.57	1.46	0.11
Cys	12.93	12.40	12.28	0.60	0.53
Asp	0.46	0.45	0.45	0.01	0.64
TNAA^d^	596.33	622.92	605.69	25.53	0.58
TFAA^d^	1166.11^b^	1256.17^a^	1182.25^ab^	29.41	0.02

^a,b^Different superscripts within a column represent significant differences (*P* < 0.05)

^c^Control, saline; Arg group, saline + 9.42 g/L *L*-Arg; Ala group: saline + 19.31 g/L *L*-Ala

^d^TEAA: Total essential amino acids; TNAA: Total non-essential amino acids; TFAA: total free amino acids

### Effects of arginine infusion on gene expression of amino acid transporters

Effects of different treatments on gene expression of amino acid transporters are described in Table 8. The infusion of Arg led to greater expression of *SLC7A2* and *SLC7A8* compared with the Control (*P* = 0.02 and *P* < 0.001 respectively) or Ala group (*P* = 0.003 and *P* < 0.001 respectively). However, no difference in *SLC7A2* and *SLC7A8* expression was found between the Control and Ala group (*P* = 0.68 and *P* = 0.25 respectively). Furthermore, there was no difference in gene expression of *SLC7A1, SLC7A6, SLC7A7* and *SLC7A5* among different groups.

**Table 8:** Effects of arginine infusion on gene expression of amino acid carriers in mammary gland (fold-change relative to control 2^−ΔΔCt^)

Genes	Treatments^c^			SEM	*P*-value
	Control	Arg group	Ala group		
*SLC7A1*	1.07	1.55	1.43	0.19	0.06
*SLC7A2*	0.97^b^	1.17^a^	0.91^b^	0.06	0.01
*SLC7A6*	0.98	1.07	1.13	0.06	0.06
*SLC7A7*	0.99	1.08	0.90	0.07	0.06
*SLC7A5*	1.00	0.98	1.03	0.05	0.60
*SLC7A8*	1.02^b^	2.23^a^	1.16^b^	0.09	< 0.01

^a,b^Different superscripts within a column represent significant differences (*P* < 0.05)

^c^Control, saline; Arg group, saline + 9.42 g/L *L*-Arg; Ala group: saline + 19.31 g/L *L*-Ala

Genes: SLC7A1 = solute carrier family 7 member 1; SLC7A2 = solute carrier family 7 member 2; SLC7A5 = solute carrier family 7 member 5; SLC7A6 = solute carrier family 7 member 6; SLC7A7 = solute carrier family 7 member 7; SLC7A8 = solute carrier family 7 member 8

## Discussion

### Effects of arginine infusion on milk production parameters

Milk is regarded as the main economic index of dairy production, and yield is closely associated with dietary CP and energy supply [3, 26, 27]. In the present study, although no difference was detected in feed intake among different groups, both milk yield and milk efficiency (calculated as milk yield/feed intake) were increased by the infusion of Arg compared with the Control. A similar result was reported in lactating sows supplemented with 1% Arg in the diet [12]. These results might be caused at least in part by an increase in mammary blood flow [28] induced by nitric oxide (NO) which is a potent vasorelaxant of the mammary vasculature [29]. Just as reported in a previous study, the infusion of Arg to the dairy cows treated with Nω-hydroxy-nor-*L*-arginine (arginase inhibitor) increased the milk yield together with NO concentration in blood [24]. However, no differences of milk fat and solids nonfat were found among different treatments. The similar results were also reported by Zhao et al. [30].

Furthermore, the infusion of Arg increased both the milk protein content and daily milk protein production of experimental cows compared with those infused with saline. At similar feed intake, the concentration of milk protein in primiparous sows fed a maintenance diet containing Arg also increased above control sows [31]. Despite its status as “semi essential AA” the fact that removal of Arg from a perfusate containing essential AA led to lower protein content in lactating cows underscores its importance [32]. From a mechanistic standpoint, the in vivo data agree with in vitro results indicating that supply of Arg to BMEC enhanced casein synthesis through activation of cellular signaling mechanisms converging on mTOR [13]. Taken the available data together, the positive effect of Arg supply on milk yield and protein synthesis likely was caused by a combination of greater blood flow via NO (another metabolite of Arg) [28], and direct effects of Arg and polyamines (metabolites of Arg) on mTOR [33, 34]. While there was a study reported that the postruminal supplementation of N also increases the milk yield and milk protein content in Friesian cows [35]. Therefore, the Ala group was included to supply equal N via jugular vein compared with Arg group in this study. The milk yield and protein concentration in Ala group were similar to those in the Control but lower than in Arg group, which helps to eliminate this query and verify the hypothesis that Arg plays important roles in the milk synthesis in addition to work as the basic unit of protein synthesis.

### Effects of arginine infusion on feed intake and the aspects of nitrogen absorption and utilization

In non-ruminants, there is some evidence that Arg affects feed intake [36, 37] through the regulation of NO action on the central nervous system [38]. However, no effect of Arg infusion on feed intake was found in present study. The similar result was also reported by Yao et al. [14] that the dietary supplementation of Arg has no effect on feed intake in neonatal pigs. Responses to enhanced supply of Arg on feed intake might be species specific and dose-depedent.

Despite the lack of effect on total daily N intake, daily production of milk protein N was highest in the Arg group. Therefore, the NUE increased to 27.53% (the highest) by infusion of Arg. In cows fed silage and concentrates, previous studies have concluded that quantity of excreted N is negatively correlated with NUE [39, 40]. In the current study, the output of urea N in urine which was reported to be positively correlated with urinary N [41] decreased by Arg infusion compared with alanine infusion. In addition, the urea in blood which is the major end product of AA oxidation in mammals [42] and suggested to be an indicator of whole body NUE [43] was also lower by infusing Arg compared with the Control and Ala group. Similarly, the output of urea N in milk (another NUE indicator closely related to urinary N) [6] was also lowest in cows infused with Arg. All these results were in line with results from sows in which dietary Arg supplementation increased N utilization and reduced plasma level of urea and output of urinary N [12].

### Effects of arginine infusion on plasma profiles and arteriovenous difference of free amino acids

A previous study with dairy cows reported that an increase of 8% TFAA in duodenal digesta resulted in a 5% increase in milk protein content [44]. In addition to the effects of enhancing TFAA supply to mammary gland, changes in the supplementation of some essential AA (lysine, methionine) also elicit effects on the production of milk protein [11, 45]. In the current study, although the concentrations of most free AA, TFAA, TEAA and TNAA were not affected by treatments, the increase of Arg concentration in coccygeal artery after Arg infusion and arterial alanine concentration after alanine infusion was consistent with a previous study [30]. As reported in sows, the Arg uptake by the mammary gland is much greater than milk Arg output [46], which indicates a high capacity of the mammary gland to catabolize Arg for other functions (regulating mammary gland blood flow, protein synthesis, fatty acid synthesis and lactogenesis) related to milk synthesis [47].

An interesting result was observed by Mateo et al. [12] that the elevated plasma Arg decreased the plasma concentrations of Ser, His, Thr and Glu but increased concentration of total AA (primarily protein) in milk. They surmised these results were caused by the increased uptake of AA by mammary gland (through a combination of greater growth of mammary gland and the blood flow to it) which induced by Arg supplementation. Infusion of Arg in the present study led to greater mammary uptake of His, Thr, Glu together with Met, Arg, TEAA and TFAA compared with the Control. However, the uptake of His, Thr, Glu, Met, Arg, TEAA and TFAA were not affected by infusion of Ala. These data agree with the study by Mateo et al. [12], and suggest that addition of Arg elicits multifaceted effects on all of which lead to greater milk protein synthesis in dairy cows.

### Effects of arginine infusion on gene expression of amino acid transporters

It has been reported that the amounts of essential AA supplied to mammary gland play important roles in the synthesis and secretion of milk protein [48]. Amino acids which are a kind of micro-molecules need to combine with the amino acid carrier proteins to get through cytomembrane. Thus, the amount and the activation of the amino acid carriers are positively correlated to the protein synthesis [49]. In present study, the gene expression of *SLC7A1*, *SLC7A5, SLC7A6* and *SLC7A7* was not affected by different treatments. However, the gene expression of *SLC7A2* and *SLC7A8* was greater in the cows infused with Arg compared with the Control or Ala group. The similar results were also reported by Ding et al. [24] that the infusion of Nω-hydroxy-nor-*L*-arginine (an arginase inhibitor) together with Arg increased the *SLC7A8* expression in bovine mammary gland*.* Furthermore, the study in porcine intestinal epithelial cells also indicated that the supplementation of Arg in culture media increased the expression of *SLC7A8* [50]*.* According to the classification method on the basis of the specifics of amino acids, SLC7A2 and SLC7A8 are two important acidic AA transporters for Arg, Lys and His. The increased expression of *SLC7A2* and *SLC7A8* might partly contribute to the increased uptake of Arg and His, and the protein synthesis in mammary gland. In addition to working as the transporter of AA, there have been some studies [51, 52] found that the amino acid carriers proton-assisted amino acid transporter, SLC7A2 and SLC7A8 are positively correlated to the mammalian target of rapamycin (mTOR) kinase which is crucial to the cell growth and proliferation, and protein synthesis [53]. Just as described in the study of Zeng et al. [50], the addition of Arg increased *SLC7A8* expression and activated mTOR, resulting in the increased growth and proliferation of intestinal epithelial cells. Although the expression of mTOR was not compared in this study, the previous study in BMEC found the increased availability of Arg promote the casein synthesis by activating mTOR [13]. Thus, the effects of Arg infusion on milk production may be related to the amino acid transporters (SLC7A2 and SLC7A8) together with mTOR.

## Conclusions

Enhancing the post-ruminal supply of Arg can have a positive effect on milk yield and protein synthesis. A number of potential direct and indirect effects (the changes in amino acid transporters and mTOR, and the blood flow) appear responsible for these effects. Further research is warranted to identify the better underlying mechanisms that N utilization efficiency can be enhanced.

## Abbreviations

AA: Amino acids
BMEC: Bovine mammary epithelial cells
CP: Crude protein
GAPDH: Glyceraldehyde-3-phosphate dehydrogenase
N: Nitrogen
NO: Nitric oxide
NUE: Nitrogen utilization efficiency
SLC7A1: Solute carrier family 7 member 1
SLC7A2: Solute carrier family 7 member 2
SLC7A5: Solute carrier family 7 member 5
SLC7A6: Solute carrier family 7 member 6
SLC7A7: Solute carrier family 7 member 7
SLC7A8: Solute carrier family 7 member 8
TEAA: Total essential amino acids
TFAA: Total free amino acids
TNAA: Total non-essential amino acids

## References

[1] Raun WR, Johnson GV (1999). Improving nitrogen use efficiency for cereal production. Agron J.

[2] Yan T, Frost JP, Agnew RE, Binnie RC, Mayne CS (2006). Relationships among manure nitrogen output and dietary and animal factors in lactating dairy cows. J Dairy Sci.

[3] Broderick GA (2003). Effects of varying dietary protein and energy levels on the production of lactating dairy cows. J Dairy Sci.

[4] Van Horn HH, Wilkie AC, Powers WJ, Nordstedt RA (1994). Components of dairy manure management systems. J Dairy Sci.

[5] Wang C, Liu JX, Zhai SW, Lai JL, Wu YM (2008). Effects of rumen-degradable-protein to rumen-undegradable-protein ratio on nitrogen conversion of lactating dairy cows. Acta Agr Scan B-S P.

[6] Hynes DN, Stergiadis S, Gordon A, Yan T (2016). Effects of crude protein level in concentrate supplements on animal performance and nitrogen utilization of lactating dairy cows fed fresh-cut perennial grass. J Dairy Sci.

[7] Zimmerman CA, Rakes AH, Jaquette RD, Hopkins BA, Croom WJ (1991). Effects of protein level and forage source on milk production and composition in early lactation dairy cows 1. J Dairy Sci.

[8] Noftsger S, St-pierre NR (2003). Supplementation of methionine and selection of highly digestible rumen undegradable protein to improve nitrogen efficiency for milk production. J Dairy Sci.

[9] Wright TC, Moscardini S, Luimes PH, Susmel P, Mcbride BW (1998). Effects of rumen-undegradable protein and feed intake on nitrogen balance and milk protein production in dairy cows. J Dairy Sci.

[10] Varvikko T, Vanhatalo A, Jalava T, Huhtanen P (1999). Lactation and metabolic responses to graded abomasal doses of methionine and lysine in cows fed grass silage diets. J Dairy Sci.

[11] Wang C, Liu HY, Wang YM, Yang ZQ, Liu JX, Wu YM (2010). Effects of dietary supplementation of methionine and lysine on milk production and nitrogen utilization in dairy cows. J Dairy Sci.

[12] Mateo RD, Wu G, Moon HK, Carroll JA, Kim SW (2008). Effects of dietary arginine supplementation during gestation and lactation on the performance of lactating primiparous sows and nursing piglets. J Anim Sci.

[13] Wang M, Xu B, Wang H, Bu D, Wang J, Loor JJ (2014). Effects of arginine concentration on the in vitro expression of casein and mTOR pathway related genes in mammary epithelial cells from dairy cattle. PLoS One.

[14] Yao K, Yin YL, Chu W, Liu Z, Deng D, Li T (2008). Dietary arginine supplementation increases mTOR signaling activity in skeletal muscle of neonatal pigs. J Nutr.

[15] Edmonson AJ, Lean IJ, Weaver LD, Farver T, Webster G (1989). A body condition scoring chart for Holstein dairy cows. J Dairy Sci.

[16] NRC (2001). Nutrient requirements of dairy cattle.

[17] Kopelove A, Hach C, Bowden B, Brayton S (1987). More powerful peroxide kjeldahl digestion method. AOAC.

[18] Lee C, Hristov AN (2013). Short communication: evaluation of acid-insoluble ash and indigestible neutral detergent fiber as total-tract digestibility markers in dairy cows fed corn silage-based diets. J Dairy Sci.

[19] Cant J, DePeters E, Baldwin R (1993). Mammary amino acid utilization in dairy cows fed fat and its relationship to milk protein depression. J Dairy Sci.

[20] Lascano GJ, Zanton GI, Heinrichs AJ, Weiss WP (2010). Technical note: a noninvasive urine collection device for female cattle: modification of the urine cup collection method. J Dairy Sci.

[21] Freudenberger DO, Burns CJ, Toyokawa K, Barry TN (1994). Digestion and rumen metabolism of red clover and perennial ryegrass/white clover forages by red deer. J Agr Sci.

[22] Bionaz M, Loor JJ (2007). Identification of reference genes for quantitative real-time PCR in the bovine mammary gland during the lactation cycle. Physiol Genomics.

[23] Loor JJ, Dann HM, Guretzky NA, Everts RE, Oliveira R, Green CA (2006). Plane of nutrition prepartum alters hepatic gene expression and function in dairy cows as assessed by longitudinal transcript and metabolic profiling. Physiol Genomics.

[24] Ding LY, Chen LM, Wang MZ, Zhang J, Loor JJ, Zhou G (2018). Inhibition of arginase via jugular infusion of N ω -hydroxy-nor- l -arginine inhibits casein synthesis in lactating dairy cows. J Dairy Sci.

[25] Livak KJ, Schmittgen TD (2001). Analysis of relative gene expression data using real-time quantitative PCR and the 2 ^−ΔΔ C T^ method. Methods.

[26] Ipharraguerre IR, Clark JH (2005). Impacts of the source and amount of crude protein on the intestinal supply of nitrogen fractions and performance of dairy cows. J Dairy Sci.

[27] Renaudeau D, Lebreton Y, Noblet J, Dourmad JY (2002). Measurement of blood flow through the mammary gland in lactating sows: methodological aspects.(abstract). J Anim Sci.

[28] Jobgen WS, Fried SK, Fu WJ, Meininger CJ, Wu G (2006). Regulatory role for the arginine–nitric oxide pathway in metabolism of energy substrates. J Nutr Biochem.

[29] Lacasse P, Farr VC, Davis SR, Prosser CG (1996). Local secretion of nitric oxide and the control of mammary blood flow. J Dairy Sci.

[30] Zhao FF, Wu TY, Wang HR, Ding LY, Ahmed G, Li HW (2018). Jugular arginine infusion relieves lipopolysaccharide-triggered inflammatory stress and improves immunity status of lactating dairy cows. J Dairy Sci.

[31] Wu G, Knabe DA (1994). Free and protein-bound amino acids in sow's colostrum and milk. J Nutr.

[32] Tian W, Wang HR, Wu TY, Ding LY, Zhao R, Khas E (2016). Milk protein responses to balanced amino acid and removal of leucine and arginine supplied from jugular-infused amino acid mixture in lactating dairy cows. J Anim Physiol Anim Nutr.

[33] Wang MZ, Ding LY, Wang C, Chen LM, Loor JJ, Wang HR (2017). Short communication: arginase inhibition reduces the synthesis of casein in bovine mammary epithelial cells. J Dairy Sci.

[34] Kong X, Wang X, Yin Y, Li X, Gao H, Bazer FW (2014). Putrescine stimulates the mTOR signaling pathway and protein synthesis in porcine trophectoderm cells1. Biol Reprod.

[35] Orskov ER, Grubb DA, Kay RN (1977). Effect of postruminal glucose or protein supplementation on milk yield and composition in Friesian cows in early lactation and negative energy balance. Brit J Nutr.

[36] Costello MJ, Morris JG, Rogers QR (1980). Effect of dietary arginine level on urinary orotate and citrate excretion in growing kittens. J Nutr.

[37] Morley JE, Flood JF (1991). Evidence that nitric oxide modulates food intake in mice. Life Sci.

[38] Toda N, Ayajiki K, Okamura T (2005). Nitric oxide and penile erectile function. Pharmacol Therapeut.

[39] Marini JC, Van Amburgh ME (2005). Partition of nitrogen excretion in urine and the feces of Holstein replacement heifers. J Dairy Sci.

[40] Burke F, O'Donovan MA, Murphy JJ, O'Mara FP, Mulligan FJ (2008). Effect of pasture allowance and supplementation with maize silage and concentrates differing in crude protein concentration on milk production and nitrogen excretion by dairy cows. Livest Sci.

[41] Marini JC, Klein JD, Sands JM, Van Amburgh ME (2004). Effect of nitrogen intake on nitrogen recycling and urea transporter abundance in lambs. J Anim Sci.

[42] Meijer AJ, Lamers WH, Chamuleau RA (1990). Nitrogen metabolism and ornithine cycle function. Physiol Rev.

[43] Coma J, Carrion D, Zimmerman DR (1995). Use of plasma urea nitrogen as a rapid response criterion to determine the lysine requirement of pigs. J Anim Sci.

[44] Volden H (1999). Effects of level of feeding and ruminally undegraded protein on ruminal bacterial protein synthesis, escape of dietary protein, intestinal amino acid profile, and performance of dairy cows. J Anim Sci.

[45] Appuhamy JADRN, Knapp JR, Becvar O, Escobar J, Hanigan MD (2011). Effects of jugular-infused lysine, methionine, and branched-chain amino acids on milk protein synthesis in high-producing dairy cows. J Dairy Sci.

[46] Trottier NL, Shipley CF, Easter RA (1997). Plasma amino acid uptake by the mammary gland of the lactating sow. J Anim Sci.

[47] O'Quinn PR, Knabe DA (2002). Wu G. arginine catabolism in lactating porcine mammary tissue. J Anim Sci.

[48] Bequette BJ, Backwell FR, Crompton LA (1998). Current concepts of amino acid and protein metabolism in the mammary gland of the lactating ruminant. J Dairy Sci.

[49] Hundal HS, Taylor PM (2009). Amino acid transceptors: gate keepers of nutrient exchange and regulators of nutrient signaling. Am J Physiol Endocrinol Metab.

[50] Zeng L, Tan B, Yin Y, Kong X, Fen Z, Fang J (2012). Gene expression profiles in porcine intestinal epithelial cells treated with arginine using a microarray technique. J. Food. Agric Environ.

[51] Nicklin P, Bergman P, Zhang B, Triantafellow E, Wang H, Nyfeler B (2009). Bidirectional transport of amino acids regulates mTOR and autophagy. Cell.

[52] Heublein S, Kazi S, Ögmundsdóttir MH, Attwood EV, Kala S, Boyd CAR (2010). Proton-assisted amino-acid transporters are conserved regulators of proliferation and amino-acid-dependent mTORC1 activation. Oncogene.

[53] Sarbassov DD, Ali SM, Sabatini DM (2005). Growing roles for the mTOR pathway. Curr Opin Cell Biol.

